# An approach for collaborative development of a federated biomedical knowledge graph-based question-answering system: Question-of-the-Month challenges

**DOI:** 10.1017/cts.2023.619

**Published:** 2023-09-14

**Authors:** Karamarie Fecho, Chris Bizon, Tursynay Issabekova, Sierra Moxon, Anne E. Thessen, Shervin Abdollahi, Sergio E. Baranzini, Basazin Belhu, William E. Byrd, Lawrence Chung, Andrew Crouse, Marc P. Duby, Stephen Ferguson, Aleksandra Foksinska, Laura Forero, Jennifer Friedman, Vicki Gardner, Gwênlyn Glusman, Jennifer Hadlock, Kristina Hanspers, Eugene Hinderer, Charlotte Hobbs, Gregory Hyde, Sui Huang, David Koslicki, Philip Mease, Sandrine Muller, Christopher J. Mungall, Stephen A. Ramsey, Jared Roach, Irit Rubin, Shepherd H. Schurman, Anath Shalev, Brett Smith, Karthik Soman, Sarah Stemann, Andrew I. Su, Casey Ta, Paul B. Watkins, Mark D. Williams, Chunlei Wu, Colleen H. Xu, The Biomedical Data Translator Consortium

**Affiliations:** 1 Renaissance Computing Institute (RENCI), University of North Carolina at Chapel Hill, Chapel Hill, NC, USA; 2 Copperline Professional Solutions, Pittsboro, NC, USA; 3 Department of Biomedical Informatics, University of Colorado Anschutz Medical Campus, Aurora, CO, USA; 4 Biosystems Data Science Department, Lawrence Berkeley National Laboratory, Berkeley, CA, USA; 5 Division of Preclinical Innovation, National Center for Advancing Translational Sciences, National Institutes of Health, Rockville, MD, USA; 6 Department of Neurology, Weill Institute for Neuroscience, University of California - San Francisco, San Francisco, CA, USA; 7 Institute for Systems Biology, Seattle, WA, USA; 8 The Hugh Kaul Precision Medicine Institute, University of Alabama at Birmingham, Birmingham, AL, USA; 9 The Broad Institute of MIT and Harvard, Cambridge, MA, USA; 10 National Institute of Environmental Health Sciences, National Institutes of Health, Research Triangle Park, NC, USA; 11 Rady Children’s Institute for Genomic Medicine, Rady Children’s Hospital, San Diego, CA, USA; 12 University of California at San Diego, San Diego, CA, USA; 13 Gladstone Institutes, University of California - San Francisco, San Francisco, CA, USA; 14 Tufts Clinical and Translational Science Institute, Tufts Medical Center, Boston, MA, USA; 15 Thayer School of Engineering at Dartmouth College, Hanover, NH, USA; 16 Departments of Computer Science and Engineering, Biology, and the Huck Institutes of the Life Sciences, Penn State University, University Park, PA, USA; 17 Swedish Medical Center, St. Joseph Health, Seattle, WA, USA; 18 University of Washington, Seattle, WA, USA; 19 Oregon State University, Corvallis, OR, USA; 20 National Institute on Aging, National Institutes of Health, Baltimore, MD, USA; 21 The Scripps Research Institute, La Jolla, CA, USA; 22 Columbia University Irving Medical Center, New York, NY, USA; 23 Division of Pharmacotherapy and Experimental Therapeutics, Eshelman School of Pharmacy, University of North Carolina at Chapel Hill, Chapel Hill, NC, USA

**Keywords:** Translational research, team science, knowledge graphs, bioinformatics, semantic technology

## Abstract

Knowledge graphs have become a common approach for knowledge representation. Yet, the application of graph methodology is elusive due to the sheer number and complexity of knowledge sources. In addition, semantic incompatibilities hinder efforts to harmonize and integrate across these diverse sources. As part of The Biomedical Translator Consortium, we have developed a knowledge graph–based question-answering system designed to augment human reasoning and accelerate translational scientific discovery: the Translator system. We have applied the Translator system to answer biomedical questions in the context of a broad array of diseases and syndromes, including Fanconi anemia, primary ciliary dyskinesia, multiple sclerosis, and others. A variety of collaborative approaches have been used to research and develop the Translator system. One recent approach involved the establishment of a monthly “Question-of-the-Month (QotM) Challenge” series. Herein, we describe the structure of the QotM Challenge; the six challenges that have been conducted to date on drug-induced liver injury, cannabidiol toxicity, coronavirus infection, diabetes, psoriatic arthritis, and *ATP1A3*-related phenotypes; the scientific insights that have been gleaned during the challenges; and the technical issues that were identified over the course of the challenges and that can now be addressed to foster further development of the prototype Translator system. We close with a discussion on Large Language Models such as ChatGPT and highlight differences between those models and the Translator system.

## Introduction

Knowledge graphs (KGs) have become a common approach for knowledge representation in numerous scientific disciplines [1]. The basic unit of knowledge representation in a KG is the “triple,” or “subject–predicate–object” relationship, in which the subject and the object are represented as nodes or core entities within a graph and the predicate is represented as an edge or a defined relationship between the subject and the object nodes. Nodes are typically mapped to preestablished ontologies; edges can be undirected, directed, or bidirectional; both nodes and edges in a KG can be qualified and annotated to support complex semantics. KGs support Wikipedia’s central data storage (i.e., Wikidata), Google’s search engine, and Amazon’s product graph, to provide a few well-known examples.

One major challenge with biomedical KGs is the breadth and diversity of available knowledge or data sources and the semantic incompatibilities that hinder efforts to harmonize and integrate across them. The Biomedical Data Translator Consortium has developed a biomedical KG-based “Translator” system designed to leverage biomedical knowledge through semantic harmonization across disparate data sets and the translation of those data into scientific insights [2,3]. The Translator system is in its fourth year of development, having first demonstrated feasibility. The system has been applied to answer subject matter expert (SME)–informed questions across diverse use cases, including Fanconi anemia, asthma, primary ciliary dyskinesia, multiple sclerosis, and many others.

The Translator Consortium has implemented numerous approaches for research, development, and testing of the Translator system [4,5]. This special communication describes one novel approach for collaborative testing of the Translator system in the context of SME-informed questions: the Question-of-the-Month (QotM) Challenge series. We describe the structure of the QotM Challenges, provide an overview of the six challenges that have been conducted to date (topics were drug-induced liver injury, cannabidiol (CBD) toxicity, coronavirus infection, diabetes, psoriatic arthritis, and *ATP1A3*-related phenotypes), and highlight the benefits of this approach for simultaneously supporting the generation of scientific insights and the identification of technical gaps and weaknesses in the prototype Translator system. We close with a discussion on Large Language Models (LLMs) such as ChatGPT and provide a comparison with the Translator system.

## Materials and Methods

The Translator system is designed to support biomedical discovery through an informatics platform that enables exploration and reasoning over an open-source, federated KG-based ecosystem, which includes > 300 integrated and harmonized data sources, largely from curated and trusted databases such as DrugBank [6] and the Comparative Toxicogenomics Database [7], and ontologies such as Monarch Disease Ontology [8] and Gene Ontology [9]. The architecture of the Translator system is complex but consists of four primary component types: an Automated Relay System (ARS); five Autonomous Relay Agents (ARAs); dozens of knowledge providers (KPs); and a Standards and Reference Implementation (SRI) component [2,3]. The role of the SRI is to create the standards and services necessary to integrate, harmonize, and communicate across Translator components. This includes the development of a communication standard – the Translator Reasoner Application Programming Interface (TRAPI) [10] – to support cross-component communication and the adoption of Biolink Model [11] as a universal schema and upper-level data model to define biomedical entities and the relationships between them. The SRI provides additional services to normalize biomedical entities that are derived from different data sources and expressed in distinct formats but that share the same semantic meaning. The ARS serves as the central hub for all Translator communication, receiving user queries in the form of TRAPI messages, transmitting the messages to the ARAs, and compiling results for presentation back to the user. The ARAs receive messages from the ARS, transmit them to KPs, and then apply a variety of reasoning and inference algorithms to KP-derived knowledge in order to find connections between biomedical entities and derive new knowledge. The KPs contribute curated domain-specific “knowledge” derived from a variety of data sources and representing either “raw” data or abstracted information. Together, the federated Translator system is capable of deriving known or novel answers to user queries.

The Translator program is highly collaborative and innovative in its approach to research and development of the Translator system. The program’s success has been attributed, in part, to the unique culture and sense of community that have enabled > 200 team members from 17 teams and dozens of institutions to work together toward a common goal [12]. One recent effort for collaborative research and development involved a monthly QotM Challenge series, driven by SME-contributed, biomedical research questions, for which answers were either unknown or only partially known.

The idea for the QotM initiative originated with a need to formally engage SMEs with the full consortium in order to accelerate research and development of the Translator system, by providing a “reality check” on the content, accuracy, and quality of Translator answers. While individual teams were engaging SMEs to research and develop their own Translator tools and services, and while the Translator Consortium periodically invited SMEs to project meetings in order to solicit their input, the consortium did not have a consistent mechanism in place for continuous SME engagement and coordinated consortium-wide testing of the Translator system. Moreover, without a user-friendly Translator user interface (UI; a prototype is under development), independent SME engagement was not feasible. Thus, the QotM Challenge series was implemented to address the need for consortium-coordinated, SME-driven research and development of the Translator system.

**Figure 1.**
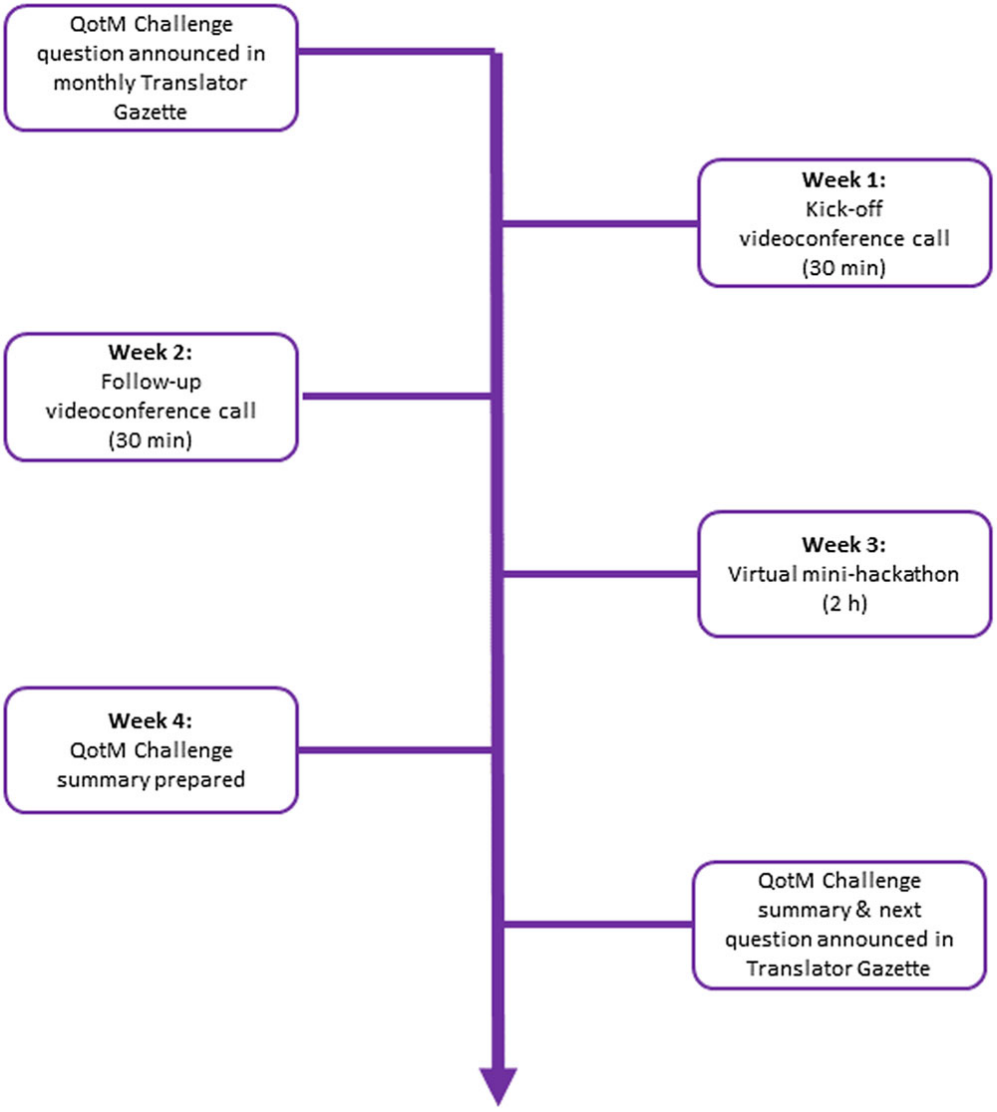
Structure of the question-of-the-month (QotM) challenge series. h = hours; min = minutes.

The QotM Challenge series is led by a moderator and structured as follows (Fig. 1). Translator team members submit potential QotM Challenges to the QotM moderator, who then works with the submitter and their QotM SME to schedule the challenge after first confirming SME availability for participation. The question is posed in natural language, and the SME provides a brief (typically one paragraph) background summary to provide context to the question. The Translator Consortium relies heavily on GitHub for both software development and project management. The QotM Challenge series is no exception, and for each QotM, the moderator creates a new issue within a shared GitHub repository. The issue contains: the name and team affiliation of the submitter; the name, academic background, and scientific interests of the SME; the specific challenge question; additional background, including links to relevant resources when provided; and the schedule for the monthly challenge. The QotM Challenge is then announced in the Translator Gazette, which is a monthly internal publication that supports cross-consortium communication and coordination, provides a forum for teams to submit short pieces describing a new tool or service, and serves as a general announcement board.

Consortium members then convene virtually via Zoom during week one for a 30-minute kick-off videoconference to brainstorm ideas and approaches for tackling the question, including approaches for translating the SME’s natural language question to TRAPI queries that Translator can respond to. Ideally, the QotM SME is present during the kick-off meeting so that Translator team members can ask additional questions related to the challenge question. Translator team members work asynchronously on the challenge until convening again for a second 30-minute videoconference during week two to provide brief updates and report on technical challenges. Any updates, blockers, or answers that have been posted to the QotM GitHub issue tracker are also reviewed. Importantly, technical issues that have been posted to the GitHub repository or that arise during the kick-off call are triaged appropriately and assigned owners. Translator team members continue working asynchronously on the challenge until convening again during week three for a two-hour virtual “mini-hackathon.” The mini-hackathon is intended to provide a focused forum to synchronously run queries, collectively troubleshoot, iteratively refine queries, and evaluate answers. The QotM SME attends all or part of the mini-hackathon in order to actively guide the direction of query development towards their scientific interests and participate in the scientific evaluation of answers. The monthly challenge culminates during week four with the QotM moderator working with the QotM SME and the submitting team to prepare a formal summary of scientific insights that were gleaned and technical issues that surfaced over the course of the challenge, including links to GitHub issues. The QotM summary is published in the Translator Gazette on Friday of week four. The new QotM Challenge is announced in the same edition.

## Results

### Overview of QotM Challenges

To date, six QotM Challenges have been conducted, each focused on a different biomedical discipline and a distinct question (Table 1). The academic background and professional interests of each SME likewise varied. Each question engaged one to three SMEs, with each SME either tangentially related to or completely unaffiliated with the Translator program. Feedback was captured in a GitHub repository and also by way of meeting recordings. A semi-formal qualitative analysis of results was conducted by core Translator team members and the SME(s) affiliated with each question.

**Table 1.** Overview of the QotM challenges

QotM Challenge	SME	Challenge Question
QotM #1	PhD; Senior Scientist, molecular toxicology and genomics	What mechanism might explain the strong association between valproic acid and *ALAS1* in *in vitro* liver models?
QotM #2	MD, PhD; Professor, molecular and cell biology, theoretical biology	What mechanism might explain the association between high levels of β-sitosterol and severe coronavirus infection?
QotM #3	MD; Professor, medicine, toxicology, and experimental therapeutics	What biological mechanisms might explain the observation that CBD is generally safe except in patients taking valproic acid?
QotM #4	MD; Professor, endocrinology, diabetes, and metabolism	What is the molecular target of an antidiabetic small molecule?*
QotM #5	MD; Practicing Physician and Clinical Professor, internal medicine and rheumatology	What are risk factors for progressing from PsO to PsA? More specifically, in a population of patients with PsO, what risk factors could be used to recruit a cohort of patients with increased risk for new onset of PsA within three years?
QotM #6	MD; Vice President for Research and Clinical Management MD; Professor, neurosciences and pediatrics MD; Fellow, genetics	Given a mutation in gene *ATP1A3* and a case description of associated phenotypes, can Translator propose new therapies?**

CBD = cannabidiol; PsA = psoriatic arthritis; PsO = psoriasis; QotM = question-of-the-month.

* Note that this question was abstracted here due to the proprietary nature of the anti-diabetic small molecule.

** Note that the specific phenotypes varied by clinical case; however, the following phenotypes were generally shared across cases, albeit with varying severity: nystagmus; episodic hemiplegia; dystonia; tremors; global developmental delay; hypotonia; seizures; gastroesophageal reflux; paroxysmal dystonia; and muscle weakness.

The two 30-minute videoconferences (weeks one and two) and the final virtual mini-hackathon were well attended. Of roughly 200 Translator team members, an average of 19 (range: 11–30) participated in the kick-off and stand-up videoconferences. (Attendance was not recorded during the mini-hackathons.) Overall, an average of seven (range: 4–10) of 17 teams total actively contributed to each QotM Challenge. Eight QotM SMEs participated in the six QotM Challenges.

### Scientific Insights Gleaned

The Translator system is designed to provide mechanistic insights into real-world laboratory and clinical observations and thereby augment human reasoning. The QotM Challenges highlighted this capability, not only in terms of the types of questions that were posed by SMEs, but also in terms of the scientific insights that were gleaned over the course of the six challenges.

**Figure 2.**
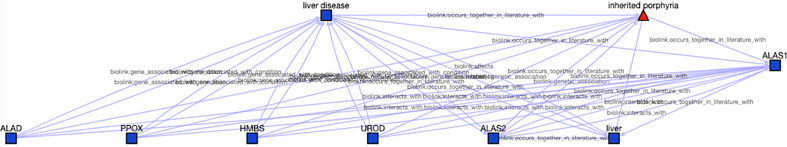
Example of a Translator answer subgraph demonstrating a relationship between liver disease and a set of genes associated specifically with inherited porphyria: *ALAS1; ALAS2*; *ALAD*; *PPOX*; *HMBS;* and *UROD*.

For instance, QotM #1 focused on an unexpected observation that valproic acid reversibly induces *ALAS1*, a gene that encodes a mitochondrial enzyme involved with heme or iron protoporphyrin biosynthesis [13], in models of liver disease or injury. The specific question was: *“What mechanism might explain the strong association between valproic acid and ALAS1 in* in vitro *liver models?”* Members of seven Translator teams actively contributed to the challenge. A variety of approaches were taken, including multi-hop queries (chained subject-predicate-object triples), graph-based enrichment analysis, and one-hop queries of Translator clinical KPs (i.e., electronic health record evidence of real-world associations). Key findings included the identification of a relationship between valproic acid, *ALAS1*, porphyria (a disease caused by overaccumulation of porphyrin, which is essential for the function of hemoglobin [14]), and the PPAR gene family [15]. Moreover, the clinical queries identified a variety of diseases and phenotypes for which valproic acid is contraindicated such as post-traumatic stress disorder, depression, and malnutrition. The SME now plans to review the evidence that Translator provided in support of these relationships and consider new experimental designs based on that evidence (Fig. 2).

QotM #2 focused on an unexplained clinical observation that patients with severe coronavirus infection also have high plasma levels of β-sitosterol, a phytosterol found in plants and not synthesized in animals [16]. The question that was posed was: *“What mechanism might explain the association between high levels of β-sitosterol and severe coronavirus infection?”* Members of nine Translator teams actively contributed to the challenge. Complex relationships were identified between β-sitosterol and cholesterol, sitosterolemia (a condition in which phytosterols accumulate in the blood and tissues [17]), soybean oil, and propofol (a sedative often given to patients prior to intubation and mechanical ventilation [18]). A Translator team member then identified an external data source, DailyMed [19], that provides structured product labels and found that the entry for propofol includes soybean oil in the “Ingredients and Appearance” section. The challenge culminated in a conceptual model to explain the observed relationship between severe coronavirus infection and β-sitosterol: severe coronavirus infection is associated with acute respiratory distress syndrome, which is treated with intubation / mechanical ventilation, for which propofol is administered as a sedative and prepared as an emulsion with soybean oil, which contains β-sitosterol.

QotM #3 focused on clinical evidence that CBD is generally safe, except in patients taking valproic acid, and specifically asked: *“What biological mechanisms might explain the observation that CBD is generally safe except in patients taking valproic acid?”* The challenge question was based on a US Food & Drug Administration (FDA) review of primary clinical trial data on EPIDIOLEX® [20], which demonstrated no signs of hepatotoxicity, *except* in children taking valproic acid. While the US FDA approved EPIDIOLEX® as the first and only approved prescription CBD, they questioned the implications of the observed hepatotoxicity of CBD when taken with valproic acid. For instance, what other drugs might show synergistic hepatotoxicity with CBD? Ten Translator teams participated in the challenge. Translator was able to replicate several published findings from the SME’s laboratory [21,22] and also gain new insights, such as a potential role for the gene *PAK1*, which is involved with cytoskeletal reorganization and nuclear signaling. The challenge concluded with the hypothesis that both valproic acid and CBD may negatively regulate *PAK1* and thereby interfere with the establishment of hepatocyte polarity, which may contribute to hepatotoxicity.

QotM #4 focused on a search for the molecular target of a novel small molecule that has demonstrated efficacy in murine models of diabetes, with an added benefit of improving fatty liver [23]. The specific question was: *“What is the molecular target of an anti-diabetic small molecule?”* Members of two Translator teams drove this challenge, using complementary but distinct approaches. This challenge was particularly interesting to Translator team members, as it required team members to consider query structures and Translator knowledge sources to explore a small molecule with very little known about it, and also balance their practices such that open team science, a Translator tenet, remained in place but was tempered to respect the proprietary nature of the challenge question. Key outcomes from this challenge included the identification of a potentially important enzyme that may help identify the molecular target of the anti-diabetic small molecule.

QotM #5 focused on the identification of risk factors (e.g., molecular hallmarks, phenotypic features) that differentiate patients with PsO who transition to PsA from those who do not. The SME’s question was two-fold: *“What are risk factors for progressing from PsO to PsA? More specifically, in a population of patients with PsO, what risk factors could be used to recruit a cohort of patients with increased risk for new onset of PsA within three years?”* The primary application of this challenge was to assist with clinical trial recruitment by allowing investigators to select a cohort enriched in patients who are likely to transition from PsO to PsA. Members of four Translator teams actively contributed to the challenge. Translator team members initially approached the challenge by seeking genes and biological pathways that are specific to PsA, but not PsO. An exclusion list of genes that Translator identified as shared by PsA and PsO was compared to a SME-generated list of genes that are known or suspected to contribute to PsA. A quick comparison of the two lists showed that the exclusion list included many of the established PsA genes (e.g., *IL17*, *TNF*, *TRAF3IP2*), as well as additional genes (e.g., *TNIP1*, *REV3L*, *SDC1*) that were deemed worthy of further exploration. From there, Translator team members executed a series of queries designed to constrain the results, given the large number of answers. The challenge concluded with a compilation of results that are now being reviewed by the SME.

QotM #6 focused on the identification of candidate therapies for a case study of five patients with various mutations in *ATP1A3* and asked specifically: *“Given a mutation in gene ATP1A3 and a case description of associated phenotypes, can Translator propose new therapies?”* The goal was to consider the phenotypes common to each case study, including motor symptoms, developmental delays, dystonia, and occasionally seizures, and use Translator to suggest candidate therapies. While a first-line treatment, flunarizine [24], has been established, it is inconsistent in its effectiveness across cases. As such, the SMEs were seeking alternatives. Team members from six teams actively participated in this challenge. Translator team members approached the challenge using a variety of strategies and readily identified flunarizine among query responses. Novel insights included the identification of two drugs that are used to treat schizophrenia, clozapine [25] and haloperidol [26], as well as botulinum toxin [27]. All three drugs were of interest to the SMEs. However, the SMEs recommended searching safer alternatives to clozapine and haloperidol and noted that botulinum toxin, while a scientifically sound alternative, is not a practical treatment. Translator team members were charged at the close of the challenge with finding safer alternatives to clozapine and haloperidol.

### Technical Gaps and Weaknesses Identified

A total of 48 unique issues were tracked in the QotM GitHub issue tracker (mean: 8 unique issues per challenge) over the course of the six QotM Challenges. The issues were collaboratively analyzed by a Translator QotM Working Group and sorted into six general categories (Table 2): bugs; UI features; data gaps; Biolink Model [11] / TRAPI [10]; answer organizing/ordering; and answer quality. The majority of issues fell under data or attribute gaps, meaning data sources or node/edge attributes within data sources that were requested by SMEs but not available in Translator. In some cases, Translator team members were unaware of the missing data sources due, in part, to a lack of adherence of those data owners to FAIR principles [28]; in other cases, Translator team members may have been aware but reluctant to divert resources in the absence of a driving use-case question. Actions were taken to resolve all issues, largely by posting GitHub issues with assignments to shared repositories or individual team repositories, or following up on existing issues. Other actions were also taken, including adding an issue to the agenda for an upcoming Translator all-hands working meeting, bringing the issue to a Translator Committee or Working Group, and assigning one or more team members to determine whether a GitHub issue is actually needed.

**Table 2.** Technical gaps and weaknesses identified as part of the translator QotM challenge series

Issue Type	Number of Issues	Example of a Specific Issue
Bugs	6	Translator service issue. β-sitosterol is incompletely mapped in the Node Normalization [29] and Name Resolver [30] services. UMLS identifier for β-sitosterol is C0106127, but it is not mapped by Node Normalizer to CHEMBL.COMPOUND:CHEMBL:221542.
UI features	6	“Compare” and “exclude” lists. Compare lists of genes, e.g., extract those that are up/down regulated. Translator cannot directly compare two answer sets to identify overlap and distinction, e.g., “in PsA gene set but not in PsO gene set.”
Data/attribute gaps	19	WikiPathways [31] and BioPlanet [32]. Considered “trusted” by toxicologists but are not Translator knowledge sources.
Biolink Model/TRAPI	6	Drug classes and side effects. Drug classes and side effects are not supported in Translator. Biolink Model [11] “has_chemical_role” attribute was added to “ChemicalEntity” class. Plan is to tag ChemicalEntity with ChEBI roles as Biolink class, with role related to a ChemicalEntity via an edge property “has_chemical_ role.”
Answer ordering/ organizing	5	Grouping. SMEs expressed a desire to group answers by, e.g., phenotypes or chemicals. Translator does not currently support this.
Answer quality	6	SemMedDB [33]: incomplete, incorrect, or missing annotation; liberal or incorrect predicate assertions. SemMedDB sometimes provides incomplete or incorrect annotation and predicate assertions, e.g., the predicate *treat* is sometimes used too liberally for assertions that are more accurately captured with predicates such as *affects*.

ChEBI = chemical entities of biological interest; PsA = psoriatic arthritis; PsO = psoriasis; QotM = question-of-the-month; SemMedDB = semantic medline database; SME = subject matter expert; UI = user interface; UMLS = unified medical language system.

## Discussion

We describe the structure of the QotM Challenge series and the six QotM Challenges that have been conducted to date. The scientific focus of the challenges varied, but all use cases were intended to apply the Translator system to provide mechanistic insights into laboratory and clinical observations. Here, we discuss the scientific, technical, and programmatic lessons learned over the course of the QotM Challenge series. We then provide a brief discussion on Translator and LLMs such as ChatGPT, as LLMs have recently gained prominence across numerous facets of society, including biomedicine, and so deserve discussion.

### Scientific Lessons Learned

The following scientific observations were explored using Translator: an unexpected laboratory finding of a strong relationship between valproic acid and the gene *ALAS1*; a clinical observation that patients with severe coronavirus infection have high plasma levels of β-sitosterol; clinical evidence demonstrating the safety of CBD, except in patients taking valproic acid; the effectiveness of a previously unrecognized small molecule in murine models of diabetes and a search for its molecular target; a search for risk factors that might predict the clinical transition from PsO to PsA; and a quest for candidate therapies for a series of case studies involving mutations in the gene *ATP1A3*.

While the Translator system did not provide definitive conclusions for the challenge questions, the system did provide mechanistic insights that SMEs are now able to use to refine their hypotheses and form additional questions. For example, Translator returned results that connected plant-derived β-sitosterol to propofol, which is a drug used to induce anesthesia prior to intubation, thus perhaps explaining the observed association that patients with severe coronavirus also have high levels of β-sitosterol. Moreover, Translator was able to leverage real-world observations from clinical KPs to supplement mechanistic assertions. Importantly, Translator was able to replicate published findings and/or identify established treatments. For instance, Translator identified publications on valproic acid–hepatotoxicity from the laboratory of the SME who posed the QotM question. Translator likewise identified flunarizine as an established treatment for phenotypes associated with *ATP1A3* mutations. This was critical to demonstrate as the QotM questions were open-ended research questions, with no known answers or only partially known answers, and so Translator’s ability to uncover the “known,” with full evidence, provenance, and confidence in the answers, fostered SME trust in the system.

### Technical Lessons Learned

In all, nearly 50 technical issues surfaced over the six QotM Challenges. General software bugs were common and not unexpected, as the Translator system is in prototype phase. Most of the bugs were straightforward and quickly resolved. In other cases, the technical issues that surfaced directly impacted Translator’s scientific and research utility. For instance, select knowledge sources were identified that were introducing issues with answer quality. These issues were more complex than simple bugs and required lengthy discussion and creative testing of approaches to resolve the issues. Text-mined knowledge, in particular, while proving to be extremely valuable to Translator, is prone to the challenges inherent in natural language processing [34]. Translator team members are testing a variety of approaches to minimize the risk of incorrect assertions from text-mined knowledge, while also maximizing the yield from such valuable resources. Complex issues such as data and attribute gaps likewise required broader discussion and assignment to a Translator committee or working group for prioritization and consensus decision. Group consensus was important because the scientific value of any new data source needed to be weighed against the necessary diversion in resources required to ingest a new data source into Translator. In all cases, actions were taken to resolve the technical issues that surfaced over the course of the QotM Challenge series, facilitated through a newly created shared GitHub repository and a new working group charged with consortium review and resolution of technical issues.

### Programmatic Lessons Learned

Several programmatic lessons also were learned as part of the QotM Challenge series. First, prior to launch of the series, we had not developed a concrete consortium-level action plan for resolving any technical issues that might arise as part of that effort or related efforts such as testing of the prototype Translator UI. Rather, the QotM moderator documented technical issues and posted a subset to various GitHub repositories, or informally assigned team members as owners, but we did not have a centralized shared GitHub repository to post issues or a formal process for subsequent review, triage, and owner assignment. The need for such a repository quickly became apparent as the number of issues increased over the course of the QotM Challenges. We corrected the course by creating a centralized GitHub repository and establishing a new meeting series and working group for review, triage, and owner assignment.

A second programmatic lesson learned related to the structure of the QotM Challenges. We repurposed two existing meeting series that had recently ended but had not yet been deleted from calendars. While this was convenient, it forced us to adopt a meeting structure that was not necessarily a good fit for the new initiative. Related, we learned that it was challenging for the QotM SMEs to attend the full two-hour mini-hackathon and that the mini-hackathon would have worked best if it was held during the final week of the QotM Challenge. We have since corrected the course by restructuring the meeting series as four weekly one-hour meetings. We also have reduced the cadence of the QotM Challenges to provide time to prepare a summary of the findings for SME review and subsequent publication in the Translator Gazette and, importantly, resolve any technical challenges that emerge. We believe that the new meeting structure will maximize the productivity of Translator team members and accelerate research and development of the Translator system. If that proves to *not* be the case, then we will correct the course yet again.

### Translator and LLMs

LLMs such as ChatGPT [35] became widely accessible and quickly rose to prominence at the end of 2022, affecting nearly every aspect of society, including biomedicine. As such, we would be remiss if we did not respond to inevitable comparisons between Translator and LLMs. Therefore, we conducted a *post hoc* systematic comparison between Translator’s performance on the QotM and ChatGPT’s performance (see Supplemental Data).

Our results showed that ChatGPT-4’s performance on the QotM Challenge questions was generally inferior to Translator’s performance. Indeed, ChatGPT failed to provide any suggested answers to two of the six questions and was not able to find any information for a third question. Moreover, our comparison identified a number of unique aspects to Translator that set it apart from ChatGPT. Briefly, in contrast to ChatGPT, Translator: (1) is fully open and transparent; (2) relies primarily on a corpus of highly curated data sources, not unjustified assertions [36]; (3) draws on all sources of knowledge in its curated knowledge sources, including edge information derived from underlying KGs; (4) invokes Biolink Model as an upper-level ontology and data model to define biomedical entities and the relationships between them; (5) is equipped with advanced reasoning tools and algorithms designed to leverage the graph-based representation of knowledge upon which the Translator system is built, allowing users to view the level of reasoning complexity that was invoked to provide a given answer; and (6) provides full evidence, provenance, and confidence in answers. Moreover, Translator does not “hallucinate” [37] or fabricate knowledge or assertions; rather, it invokes reasoning algorithms to expose curated knowledge or draw inferences, supported by complete evidence, provenance, and confidence. In addition, Translator is not prone to variation in responses due to the nuances of “prompts” and the regeneration of answers, although as a federated system, Translator’s underlying knowledge is continually maturing and expanding and so answers derived from Translator and/or their ranking may change over time. (See Supplemental Data for additional discussion.)

Despite the weaknesses of ChatGPT, we acknowledge the potential utility of LLMs. We also recognize that LLMs might complement and even enhance Translator, and *vice versa*. For instance, Translator might benefit from ChatGPT’s natural language processing capability. Likewise, ChatGPT might benefit from Translator’s graphical representation of answers as subgraphs that explicitly describe the reasoning path and include complete evidence, provenance, and confidence in all assertions. A combination of both forms of knowledge representation may prove quite powerful. Moreover, we have been experimenting with the ability for ChatGPT to call out to Translator components via the ChatGPT-4 plugin mechanism. We also are investigating how Translator components might take advantage of GPT-4 capabilities through the OpenAI Application Programming Interface. These are but a few examples. Other opportunities are likely to emerge as we learn more about ChatGPT and other LLMs.

## Conclusion

The QotM Challenge series provided a successful forum to collaboratively engage SMEs and foster further development of the prototype Translator system. We learned valuable lessons that sometimes required course corrections and program restructuring. Overall, we believe that the series was extremely useful. We expect that our approach to collaborative software development, as well as the Translator system itself, will accelerate clinical and translational science by augmenting human reasoning, ultimately leading to more rapid improvements in human health and well-being. We encourage other large-scale consortia to consider adopting our model when researching and developing a complex technical software product.

## Supporting information

Fecho et al. supplementary material 1 Fecho et al. supplementary material

Fecho et al. supplementary material 2 Fecho et al. supplementary material

## Supplementary material

The supplementary material for this article can be found at https://doi.org/10.1017/cts.2023.619.

## Collaborative consortial authors and affiliations

Liliana Acevedo (Oregon State University, Corvallis, OR); Stanley C. Ahalt (University of North Carolina at Chapel Hill, Chapel Hill, NC); Ahmed Alkanaq (The Broad Institute of MIT and Harvard, Cambridge, MA); Ricardo Avila (The Scripps Research Institute, La Jolla, CA); Michael Bada (University of Colorado Anschutz Medical Campus, Aurora, CO); Jim Balhoff (University of North Carolina at Chapel Hill, Chapel Hill, NC); Sergio E. Baranzini (University of California at San Francisco, San Francisco, CA.); Andrew Baumgartner (Institute for Systems Biology, Seattle, WA); William Baumgartner (University of Colorado at Denver, Denver, CO); Basazin Belhu (Institute for Systems Biology, Seattle, WA); Chris Bizon (Unversity of North Carolina at Chapel Hill, Chapel Hill, NC); Namdi Brandon (CoVar Applied Technologies, Durham, NC); Matt Brush (University of Colorado Anschutz Medical Campus, Aurora, CO); Richard Bruskiewich (Star Informatics, North Sooke, British Columbia, Canada); Noel Burtt (The Broad Institute of MIT and Harvard, Cambridge, MA); William Byrd (University of Alabama at Birmingham, Birmingham, AL); Jackson Callaghan (The Scripps Research Institute, La Jolla, CA); Marco Alvarado Cano (The Scripps Research Institute, La Jolla, CA); Steven Carrell (Oregon State University, Corvallis, OR); Remzi Celebi (Maastricht University, Maastricht, Netherlands); Zhehuan Chen (Columbia University, New York, NY); Larry Chung (The Broad Institute of MIT and Harvard, Cambridge, MA); Paul A. Clemons (The Broad Institute of MIT and Harvard, Cambridge, MA); Kevin Cohen (University of Colorado at Denver, Denver, CO); Maria Costanzo (The Broad Institute of MIT and Harvard, Cambridge, MA); Andrew Crouse (University of Alabama at Birmingham, Birmingham, AL); Vlado Dančík (The Broad Institute of MIT and Harvard, Cambridge, MA); Ricardo De Miranda Azevedo (Maastricht University, Maastricht, Netherlands); Eric Deutsch (Institute for Systems Biology, Seattle, WA); Jennifer Dougherty (Institute for Systems Biology, Seattle, WA); Marc P. Duby (The Broad Institute of MIT and Harvard, Cambridge, MA); Michel Dumontier (Maastricht University, Maastricht, Netherlands); Venkata Duvvuri (Institute for Systems Biology, Seattle, WA); Stephen Edwards (RTI International, Research Triangle Park, NC); Vincent Emonet (Maastricht University, Maastricht, Netherlands); Karamarie Fecho (University of North Carolina at Chapel Hill, Chapel Hill, NC); Nathaniel Fehrmann (University of Alabama at Birmingham, Birmingham, AL); Stephen S. Ferguson (National Institute of Environmental Health Sciences, Research Triangle Park, NC); Jason Flannick (The Broad Institute of MIT and Harvard, Cambridge, MA); Alexandra M. Foksinska (University of Alabama at Birmingham, Birmingham, AL); Laura Forero (Rady Children’s Hospital, San Diego, CA; University of California at San Diego, San Diego, CA); Jennifer Friedman (Rady Children’s Hospital, San Diego, CA; University of California at San Diego, San Diego, CA); Vicki Gardner (University of North Carolina at Chapel Hill, Chapel Hill, NC); Edgar Gatica (University of Colorado at Denver, Denver, CO); Amy Glen (Oregon State University, Corvallis, OR); Gwênlyn Glusman (Institute for Systems Biology, Seattle, WA); Prateek Goel (Drexel University, Philadelphia, PA); Joseph Gormley (Tufts University, Medford, MA); Jennifer J. Hadlock (Institute for Systems Biology, Seattle, WA); Melissa A. Haendel (University of Colorado Anschutz Medical Campus, Aurora, CO); Kristina Hanspers (University of California at San Francisco, San Francisco, CA); Nomi L. Harris (Lawrence Berkeley National Laboratory, Berkeley, CA); Kaiwen He (University of Alabama at Birmingham, Birmingham, AL); Jeff Henrickson (University of Alabama at Birmingham, Birmingham, AL); Eugene W. Hinderer (Tufts Medical Center, Boston, MA); Maureen Hoatlin (Independent Contractor, Portland, OR); Charlotte A. Hobbs (Rady Children’s Hospital, San Diego, CA); Andrew Hoffman (Radboud University Nijmegen, Amsterdam, Netherlands); Conrad Huang (University of California at San Francisco, San Francisco, CA); Sui Huang (Institute for Systems Biology, Seattle, WA); Robert Hubal (University of North Carolina at Chapel Hill, Chapel Hill, NC); Lawrence Hunter (University of Colorado at Denver, Denver, CO); Greg Hyde (Dartmouth College, Hanover, NH); Tursynay Issabekova (University of Colorado Anschutz Medical Campus, Aurora, CO); Matthew Jarrell (University of Alabama at Birmingham, Birmingham, AL); Adam Johs (Drexel University, Philadelphia, PA); Jimin Kang (The Broad Institute of MIT and Harvard, Cambridge, MA); Yaphet Kebede (University of North Carolina at Chapel Hill, Chapel Hill, NC); Keum Joo Kim (Dartmouth College, Hanover, NH); Michael Knowles (University of North Carolina at Chapel Hill, Chapel Hill, NC); Ryan Koesterer (The Broad Institute of MIT and Harvard, Cambridge, MA); Daniel Korn (University of North Carolina at Chapel Hill, Chapel Hill, NC); David Koslicki (The Penn State University, University Park, PA); Ashok Krishnamurthy (University of North Carolina at Chapel Hill, Chapel Hill, NC); Lindsey Kvarfordt (Oregon State University, Corvallis, OR); Jay Lee (Columbia University, New York, NY); Jason Lin (The Scripps Research Institute, La Jolla, CA); Shaopeng Liu (The Penn State University, University Park, PA); Zheng Liu (Oregon State University, Corvallis, OR); Chunyu Ma (The Penn State University, University Park, PA); Andrew Magis (Institute for Systems Biology, Seattle, WA); Tarun Mamidi (University of Alabama at Birmingham, Birmingham, AL); Meisha Mandal (RTI International, Research Triangle Park, NC); Michelle Mantilla (The Broad Institute of MIT and Harvard, Cambridge, MA); Denise Mauldin (Institute for Systems Biology, Seattle, WA); Philip Mease (Swedish Medical Center, St. Joseph Health, Seattle, WA; University of Washngton, Seattle, WA); Luis Mendoza (Institute for Systems Biology, Seattle, WA); Abrar Mesbah (CoVar Applied Technologies, Durham, NC); Matthew Might (University of Alabama at Birmingham, Birmingham, AL); Kenny Morton (CoVar Applied Technologies, Durham, NC); Sierra A.T. Moxon (Lawrence Berkeley National Laboratory, Berkeley, CA); Sandrine Muller (The Broad Institute of MIT and Harvard, Cambridge, MA); Arun Teja Muluka (The Penn State University, University Park, PA); Christopher J. Mungall (Lawrence Berkeley National Laboratory, Berkeley, CA); John Osborne (University of Alabama at Birmingham, Birmingham, AL); Michael Patton (University of Alabama at Birmingham, Birmingham, AL); David B. Peden (University of North Carolina at Chapel Hill, Chapel Hill, NC); Alexander Pico (University of California at San Francisco, San Francisco, CA); Elizabeth Pollard (University of Alabama at Birmingham, Birmingham, AL); Guthrie Price (BMA, Leavenworth, KS); Tim Putman (University of Colorado Anschutz Medical Campus, Aurora, CO); Guangrong Qin (Institute for Systems Biology, Seattle, WA); Stephen A. Ramsey (Oregon State University, Corvallis, OR); Jason Reilly (University of North Carolina at Chapel Hill, Chapel Hill, NC); Anders Riutta (University of California at San Francisco, San Francisco, CA); Jared Roach (Institute for Systems Biology, Seattle, WA); Greg Rosenblatt (University of Alabama at Birmingham, Birmingham, AL); Irit Rubin (Institute for Systems Biology, Seattle, WA); Sienna Rucka (University of Alabama at Birmingham, Birmingham, AL); Rayn Sakaguchi (CoVar Applied Technologies, Durham, NC); Eugene Santos (Dartmouth College, Hanover, NH); Kevin Schaper (University of Colorado Anschutz Medical Campus, Aurora, CO); Shepherd Schurman (National Institue on Aging, Bethesda, MD); Anath Shalev (University of Alabama at Birmingham, Birmingham, AL); Ilya Shmulevich (Institute for Systems Biology, Seattle, WA); Shalki Shrivastava (University of North Carolina at Chapel Hill, Chapel Hill, NC); Brett Smith (Institute for Systems Biology, Seattle, WA); Karthik Soman (University of California at San Francisco, San Francisco, CA); Michael Strasser (Institute for Systems Biology, Seattle, WA); Andrew I. Su (The Scripps Research Institute, La Jolla, CA); Casey Ta (Columbia University, New York, NY); Anne E. Thessen (University of Colorado Anschutz Medical Campus, Aurora, CO); Thi Tran-Nguyen (University of Alabama at Birmingham, Birmingham, AL); Alexander Tropsha (University of North Carolina at Chapel Hill, Chapel Hill, NC); Gaurav Vaidya (University of North Carolina at Chapel Hill, Chapel Hill, NC); Luke Veenhuis (Dartmouth College, Hanover, NH); Adam Viola (CoVar Applied Technologies, Durham, NC); Max Wang (CoVar Applied Technologies, Durham, NC); Paul B. Watkins (University of North Carolina at Chapel Hill, Chapel Hill, NC); Rosina Weber (Drexel University, Philadelphia, PA); Qi Wei (Institute for Systems Biology, Seattle, WA); Chunhua Weng (Columbia University, New York, NY); Andrew Williams (Tufts University, Medford, MA); Mark D. Williams (National Center for Advancing Translational Sciences, Rockville, MD); Erica Wood (Oregon State University, Corvallis, OR); Chunlei Wu (The Scripps Research Institute, La Jolla, CA); Colleen H. Xu (The Scripps Research Institute, La Jolla, CA); Chase Yakaboski (Dartmouth College, Hanover, NH); Yao Yao (The Scripps Research Institute, La Jolla, CA); Hong Yi (University of North Carolina at Chapel Hill, Chapel Hill, NC); Arif Yilmaz (Maastricht University, Maastricht, Netherlands); Qian Zhu (National Center for Advancing Translational Sciences, Rockville, MD); Tom Zisk (Tufts University, Medford, MA).
