# Supplementary material for: An approach for collaborative development of a federated biomedical knowledge graph-based question-answering system: Question-of-the-Month challenges
Source: J Clin Transl Sci. 2023 Sep 14;7(1):e214. doi: 10.1017/cts.2023.619 (PMC10603356; doi:10.1017/cts.2023.619)
Supplement: Fecho et al. supplementary material 2 — Fecho et al. supplementary material [file S2059866123006192sup002.docx]

## *Journal of Clinical and Translational Science - Special Communication*

## *Supplemental Data*

# An Approach for Collaborative Development of a Federated Biomedical Knowledge Graph–Based Question-Answering System: Question-of-the-Month Challenges

#### Karamarie Fecho,^1,2*^ Chris Bizon,^1^ Tursynay Issabekova,^3^ Sierra Moxon,^4^ Anne E. Thessen,^3^ Shervin Abdollahi,^5^ Sergio E. Baranzini,^6^ Basazin Belhu,^7^ William E. Byrd,^8^ Lawrence Chung,^9^ Andrew Crouse,^8^ Marc P. Duby,^9^ Stephen Ferguson,^10^ Aleksandra Foksinska,^8^ Laura Forero,^11,12^ Jennifer Friedman,^11,12^ Vicki Gardner,^1^ Gwênlyn Glusman,^7^ Jennifer Hadlock,^7^ Kristina Hanspers,^13^ Eugene Hinderer,^14^ Charlotte Hobbs,^11^ Gregory Hyde,^15^ Sui Huang,^7^ David Koslicki,^16^ Philip Mease,^17,18^ Sandrine Muller,^9^ Christopher J. Mungall,^4^ Stephen A. Ramsey,^19^ Jared Roach,^7^ Irit Rubin,^7^ Shepherd H. Schurman,^20^ Anath Shalev,^8^ Brett Smith,^7^ Karthik Soman,^21^ Sarah Stemann,^5^ Andrew I. Su,^22^ Casey Ta,^23^ Paul B. Watkins,^24^ Mark D. Williams,^5^ Chunlei Wu,^22^ Colleen H. Xu;^22^ and The Biomedical Data Translator Consortium

^1^Renaissance Computing Institute (RENCI), University of North Carolina at Chapel Hill, Chapel Hill, NC 27517; ^2^Copperline Professional Solutions, Pittsboro, NC 27312; ^3^Department of Biomedical Informatics, Anschutz School of Medicine, University of Colorado, Aurora, CO 80045; ^4^Biosystems Data Science Department, Lawrence Berkeley National Laboratory, Berkeley, CA 94720; ^5^Division of Preclinical Innovation, National Center for Advancing Translational Sciences, National Institutes of Health, Rockville, MD 20850; ^6^Weill Institute for Neuroscience, Department of Neurology, University of California - San Francisco, San Francisco, CA 94158; ^7^Institute for Systems Biology, Seattle, WA 98109; ^8^The Hugh Kaul Precision Medicine Institute, University of Alabama at Birmingham, Birmingham, AL, United States 35294; ^9^The Broad Institute of MIT and Harvard University, Cambridge MA 02142; ^10^National Institute of Environmental Health Sciences, National Institutes of Health, Research Triangle Park, NC 27709; ^11^Rady Children's Institute for Genomic Medicine, Rady Children’s Hospital, San Diego, CA 92123; ^12^University of California - San Diego, San Diego, CA 92123; ^13^Gladstone Institutes, University of California - San Francisco, San Francisco, CA 94158; ^14^Tufts Clinical and Translational Science Institute, Tufts Medical Center, Boston, MA 02111; ^15^Thayer School of Engineering at Dartmouth College, Hanover, NH 03755; ^16^Departments of Computer Science and Engineering, Biology, and the Huck Institutes of the Life Sciences, Penn State University, University Park, PA 16802; ^17^Swedish Medical Center, St. Joseph Health, Seattle, WA 98109; ^18^University of Washington, Seattle, WA 98109; ^19^Oregon State University, Corvallis, OR 97331; ^20^National Institute on Aging, National Institutes of Health, Baltimore, MD 21225; ^21^Weill Institute for Neuroscience, Department of Neurology, University of California - San Francisco, San Francisco, CA 94158; ^22^The Scripps Research Institute, La Jolla, CA 92037; ^23^Columbia University Irving Medical Center, New York, NY 10032; ^24^Division of Pharmacotherapy and Experimental Therapeutics, Eshelman School of Pharmacy, University of North Carolina at Chapel Hill, Chapel Hill, North Carolina 27599

**Apart from the first five primary authors, all other primary authors are listed in alphabetical order*

+ The Biomedical Data Translator Consortium, collaborative/consortial authors

## S.1. Translator and Large Language Models (LLMs)

LLMs such as ChatGPT [1] became widely accessible and quickly rose to prominence at the end of 2022, affecting nearly every aspect of society, including biomedicine. As such, we would be remiss if we did not respond to inevitable comparisons between Translator and LLMs. Therefore, we conducted a *post hoc* systematic comparison between Translator’s performance on the Question-of-the-Month (QotM) and ChatGPT’s performance.

Specifically, we ran all six QotM questions through ChatGPT-4, reviewed responses, and summarized results (Table S1). The questions were submitted to ChatGPT-4 exactly as listed in the main manuscript (Table 1). One Translator team member was tasked with submitting the questions to ChatGPT one time only, to avoid biasing the results with repeated questions and regeneration of answers, and capturing the results. The results were then reviewed and summarized by Translator team members.

**Table S1.** Summary of ChatGPT-4’s performance on QotM Challenge questions.

| **QotM Question** | **Summary of ChatGPT-4 Response** | **ChatGPT-4 Query and Response** |
| --- | --- | --- |
| QotM #1 | ChatGPT did not offer suggested answers to the question. | <https://chat.openai.com/share/12023210-103c-4a9a-a822-117060122be6> |
| QotM #2 | ChatGPT did not offer suggested answers to the question. | <https://chat.openai.com/share/16e2daa0-54be-43f8-90a1-6455dc3ba8ab> |
| QotM #3 | ChatGPT provided the known relationship between CBD, valproic acid, and hepatotoxicity, but it did not make any suggestions for particular biological mechanisms to explain the relationship, including the insightful *PAK1* suggestion that Translator produced. | <https://chat.openai.com/share/3128de73-bc05-469d-9dbd-0f29b80ff527> |
| QotM #4 | ChatGPT had no information on the queried compound. | *N/A** |
| QotM #5 | ChatGPT offered a number of epidemiologic-type answers to the question (e.g., smoking, comorbidities), in contrast to the list of disease-specific genes produced by Translator; therefore, in this example, the types of answers produced by the two systems were very different and highly complementary. | <https://chat.openai.com/share/81678441-d4ac-4be6-a2e9-6dead28468d7> |
| QotM #6 | ChatGPT provided general population-based guidelines for *ATP1A3*-related disorders, but it did not tailor these to the list of phenotypes** that was provided. | <https://chat.openai.com/share/9dcf2428-1e80-4467-98c1-79b3871a8f8c> |

*Abbreviations: QotM = Question-of-the-Month*

**The challenge question was based on a proprietary compound and thus the response is not provided here. However, the question that was posed to both Translator and ChatGPT-4 was identical and included the name of the proprietary small molecule.*

***Note that the specific phenotypes varied by clinical case; however, the following phenotypes were generally shared across cases, albeit with varying severity: nystagmus; episodic hemiplegia; dystonia; tremors; global developmental delay; hypotonia; seizures; gastroesophageal reflux; paroxysmal dystonia; muscle weakness.*

As Table S1 indicates, ChatGPT-4’s performance was generally inferior to Translator’s performance. Moreover, our comparison identified a number of unique aspects to Translator that set it apart from ChatGPT. Specifically, Translator: (1) is fully open and transparent; (2) relies primarily on corpus of highly curated data sources, not unjustified assertions [2]; (3) draws on all sources of knowledge in its curated knowledge sources, including edge information derived from underlying KGs; (4) invokes Biolink Model as an upper-level ontology and data model to define biomedical entities and the relationships between them; (5) is equipped with advancing reasoning tools and algorithms designed to leverage the graph-based representation of knowledge upon which the Translator system is built, allowing users to view the level of reasoning complexity that was invoked to provide a given answer; and (6) provides full evidence, provenance, and confidence in answers. Moreover, Translator does not “hallucinate” or fabricate knowledge or assertions [3]; rather, it invokes reasoning algorithms to expose curated knowledge or draw inferences, supported by complete evidence, provenance, and confidence. In addition, Translator is not prone to variation in responses due to the nuances of “prompts” and the regeneration of answers, although as a federated system, Translator’s underlying knowledge is continually maturing and expanding and so answers derived from Translator and/or their ranking may change over time.

In contrast, ChatGPT: (1) is available as a free version or a subscription version equipped with advanced features, but neither version is transparent; (2) relies on unclear and questionable data sources, potentially raising concerns about licensing issues when reusing content from LLM responses; (3) trains on large amounts of text to identify probabilistic patterns and co-occurrences of terms, but lacks ontologies and other tools to accurately interpret the diverse contextual knowledge inherent in the training data, which can lead to misinterpretation of words such as “treats”; (4) does not invoke a structured data model to harmonize across entities and specify relationships between them, nor does it focus on a specific domain; (5) relies on a deep neural network architecture that is not mirroring multi-step scientific reasoning but rather is optimized for text generation independently of any scientific reasoning constraints; and (6) does not provide evidence, provenance, or confidence in answers on its own, although it can when coupled with a Bing search and/or ChatGPT plugins. In addition, ChatGPT is known to “hallucinate” [3] and is prone to variation in responses due to the nuances of prompts and the biases introduced through regeneration of answers.

Despite the weaknesses of ChatGPT, we acknowledge the potential utility of LLMs. We also recognize that LLMs might complement and even enhance Translator, and *vice versa*. For instance, Translator cannot process a user’s natural language question, unlike ChatGPT. Even the prototype Translator UI is template-based. The technology supporting ChatGPT’s natural language processing capability may be something that Translator can leverage. In addition, ChatGPT generates detailed, well-written, natural-language summaries of information. Translator provides users with a graphical representation of answers as subgraphs that explicitly describe the reasoning path and include complete evidence, provenance, and confidence in all assertions. A combination of both forms of knowledge representation may prove quite powerful. Moreover, we have been experimenting with the ability for ChatGPT to call out to Translator components via the ChatGPT-4 plugin mechanism. We also are investigating how Translator components might take advantage of GPT-4 capabilities through the OpenAI API. These are but a few examples. Other opportunities are likely to emerge as we learn more about ChatGPT and other LLMs.

**Acknowledgments.** The authors are grateful for the leadership and support provided by Dr. Tyler F. Beck, Dr. Christine M. Colvis, and the rest of the Translator Extramural Leadership Team at the National Center for Advancing Translational Sciences (NCATS). They also acknowledge the Intramural Research Program at NCATS for their support of the work described herein and the Publications Committee at NCATS for their review and approval of the manuscript for publication.

**Funding.** This work was supported by the NCATS Biomedical Data Translator Program (other transaction awards OT2TR003434, OT2TR003436, OT2TR003428, OT2TR003448, OT2TR003427, OT2TR003430, OT2TR003433, OT2TR003450, OT2TR003437, OT2TR003443, OT2TR003441, OT2TR003449, OT2TR003445, OT2TR003422, OT2TR003435, OT3TR002026, OT3TR002020, OT3TR002025, OT3TR002019, OT3TR002027, OT2TR002517, OT2TR002514, OT2TR002515, OT2TR002584, OT2TR002520; Contract number 75N95021P00636). Additional funding was provided by the Intramural Research Program at NCATS (ZIA TR000276-06).

**Disclosures.** JF receives additional funding from the Rady Children's Institute for Genomic Medicine's, and her spouse is Founder and Principal of Friedman Bioventure. JH receives grant/contract support (paid to institution) from: Pfizer; Novartis; Janssen; BMS; and Gilead. PJM receives grant/research support from: AbbVie; Amgen; Bristol Myers Squibb; Eli Lilly; Galapagos; Gilead; Janssen; Novartis; Pfizer; Sun Pharma; and UCB. PJM also serves as a consultant at: AbbVie; Acelyrin; Aclaris; Amgen; Boehringer Ingelheim; Bristol Myers Squibb; Eli Lilly; Galapagos; Gilead; GlaxoSmithKline; Inmagene; Janssen; Pfizer; Moonlake Pharma; Novartis; Sun Pharma; and UCB. In addition, PJM receives speakers’ bureau fees from: AbbVie; Amgen; Eli Lilly; Janssen; Novartis; Pfizer; and Union Chimique Belge. SS is supported by the National Institute on Aging, Intramural Research Program. All other primary authors have no conflicts of interest to declare.

## S.2. References

1. **OpenAI.** GPT-4. GPT-4 is OpenAI’s most advanced system, producing safer and more useful responses. 2015–2023. (<https://openai.com/gpt-4>) [Accessed July 10, 2023]
2. **Alston WP.** Beyond "Justification": dimensions of epistemic evaluation. Cornell University Press, Ithaca, NY 2005, ISBN 0-8014-4291-5.
3. **Ji Z, Lee N, Frieske R, et al.** Survey of hallucination in natural language generation. *ACM Computing Surveys* 2023; **55**(12): Article No. 248, pp. 1–38.
